# Supplementary material for: A fault-intrusion-tolerant system and deadline-aware algorithm for scheduling scientific workflow in the cloud
Source: PeerJ Comput Sci. 2021 Nov 2;7:e747. doi: 10.7717/peerj-cs.747 (PMC8576554; doi:10.7717/peerj-cs.747)
Supplement: Supplemental Information 6 [file peerj-cs-07-747-s006.zip › Myproject1.1/docs/org/cloudbus/cloudsim/util/WorkloadFileReader.html]

WorkloadFileReader


JavaScript is disabled on your browser.


- Overview
- Package
- Class
- Use
- Tree
- Deprecated
- Index
- Help

- Prev Class
- Next Class

- Frames
- No Frames

- All Classes

- Summary:
- Nested |
- Field |
- Constr |
- Method

- Detail:
- Field |
- Constr |
- Method


org.cloudbus.cloudsim.util

## Class WorkloadFileReader

- java.lang.Object
- - org.cloudbus.cloudsim.util.WorkloadFileReader

- All Implemented Interfaces:
  :   WorkloadModel

  ---

    

  ```
  public class WorkloadFileReader
  extends java.lang.Object
  implements WorkloadModel
  ```

  This class is responsible for reading resource traces from a file and creating a list of jobs.

  **NOTE:**
  - This class can only take one trace file of the following format: *ASCII text, zip,
    gz.*- If you need to load multiple trace files, then you need to create multiple instances of this
      class each with a unique
      entity name.- If size of the trace file is huge or contains lots of traces please increase the JVM heap
        size accordingly by using java -Xmx option when running the simulation.- The default job file size for sending to and receiving from a resource is
          `gridsim.net.Link#DEFAULT_MTU`. However, you can specify the file size by using
          `#setGridletFileSize(int)`.- A job run time is only for 1 PE not the total number of allocated PEs. Therefore, a
            Gridlet length is also calculated for 1 PE.  
            For example, job #1 in the trace has a run time of 100 seconds for 2 processors. This means each
            processor runs job #1 for 100 seconds, if the processors have the same specification.

  By default, this class follows the standard workload format as specified in 
  http://www.cs.huji.ac.il/labs/parallel/workload/   
  However, you can use other format by calling the below methods before running the simulation:
  - `setComment(String)`- `setField(int, int, int, int, int)`

  Since:
  :   5.0

  See Also:
  :   `Workload`

- - ### Constructor Summary

    Constructors

    Constructor and Description

    `WorkloadFileReader(java.lang.String fileName,
    int rating)`

    Create a new `WorkloadFileReader` object.
  - ### Method Summary

    Methods

    | Modifier and Type | Method and Description |
    | --- | --- |
    | `java.util.ArrayList<Cloudlet>` | `generateWorkload()` Reads job information from a given file. |
    | `boolean` | `setComment(java.lang.String cmt)` Identifies the start of a comment line. |
    | `boolean` | `setField(int maxField, int jobNum, int submitTime, int runTime, int numProc)` Tells this class what to look in the trace file. |

    - ### Methods inherited from class java.lang.Object

      `clone, equals, finalize, getClass, hashCode, notify, notifyAll, toString, wait, wait, wait`

- - ### Constructor Detail


    - #### WorkloadFileReader

      ```
      public WorkloadFileReader(java.lang.String fileName,
                        int rating)
                         throws java.io.FileNotFoundException
      ```

      Create a new `WorkloadFileReader` object.

      Parameters:
      :   `fileName` - the workload trace filename in one of the following format: *ASCII text,
          zip, gz.*
      :   `rating` - the resource's PE rating

      Throws:
      :   `java.io.FileNotFoundException`
      :   `java.lang.IllegalArgumentException` - This happens for the following conditions:
          - the workload trace file name is null or empty- the resource PE rating <= 0
  - ### Method Detail


    - #### generateWorkload

      ```
      public java.util.ArrayList<Cloudlet> generateWorkload()
      ```

      Reads job information from a given file.

      **Specified by:**
      :   `generateWorkload` in interface `WorkloadModel`

      Returns:
      :   the list of gridlets read from the file; `null` in case of failure.


    - #### setComment

      ```
      public boolean setComment(java.lang.String cmt)
      ```

      Identifies the start of a comment line.

      Parameters:
      :   `cmt` - a character that denotes the start of a comment, e.g. ";" or "#"

      Returns:
      :   `true` if it is successful, `false` otherwise


    - #### setField

      ```
      public boolean setField(int maxField,
                     int jobNum,
                     int submitTime,
                     int runTime,
                     int numProc)
      ```

      Tells this class what to look in the trace file. This method should be called before the
      start of the simulation.

      By default, this class follows the standard workload format as specified in 
      http://www.cs.huji.ac.il/labs/parallel/workload/   
      However, you can use other format by calling this method.

      The parameters must be a positive integer number starting from 1. A special case is where
      jobNum == -1, meaning the job or gridlet ID starts at 1.

      Parameters:
      :   `maxField` - max. number of field/column in one row
      :   `jobNum` - field/column number for locating the job ID
      :   `submitTime` - field/column number for locating the job submit time
      :   `runTime` - field/column number for locating the job run time
      :   `numProc` - field/column number for locating the number of PEs required to run a job

      Returns:
      :   `true` if successful, `false` otherwise

      Throws:
      :   `java.lang.IllegalArgumentException` - if any of the arguments are not within the acceptable ranges


- Overview
- Package
- Class
- Use
- Tree
- Deprecated
- Index
- Help

- Prev Class
- Next Class

- Frames
- No Frames

- All Classes

- Summary:
- Nested |
- Field |
- Constr |
- Method

- Detail:
- Field |
- Constr |
- Method
